# Protocol for the multicentre prospective paediatric craniectomy and cranioplasty registry (pedCCR) under the auspices of the European Society for Paediatric Neurosurgery (ESPN)

**DOI:** 10.1007/s00381-022-05540-5

**Published:** 2022-05-09

**Authors:** Thomas Beez, Martin U. Schuhmann, Paolo Frassanito, Federico Di Rocco, Ulrich W. Thomale, Hans Christoph Bock

**Affiliations:** 1 Department of Neurosurgery, Medical Faculty, Heinrich-Heine-Universität, Düsseldorf, Moorenstrasse 5, 40225 Düsseldorf, Germany; 2 Pediatric Neurosurgery, Universitätsklinikum Tübingen, Tübingen, Germany; 3 Pediatric Neurosurgery, Fondazione Policlinico Universitario A. Gemelli IRCCS, Rome, Italy; 4 Service de Neurochirurgie Pédiatrique, Hôpital Femme Mère Enfant, Lyon, France; 5 Pediatric Neurosurgery, Charité Universitätsmedizin Berlin, Berlin, Germany; 6 Department of Neurosurgery, Universitätsmedizin Göttingen, Göttingen, Germany

**Keywords:** Decompressive craniectomy, Autologous cranioplasty, Allogeneic cranioplasty, Intracranial pressure, Bone flap resorption, Functional outcome

## Abstract

**Purpose:**

In the paediatric age group, the overall degree of evidence regarding decompressive craniectomy (DC) and cranioplasty is low, whereas in adults, randomised controlled trials and prospective multicentre registries are available. To improve the evidence-based treatment of children, a consensus was reached to establish a prospective registry under the auspices of the European Society for Pediatric Neurosurgery (ESPN).

**Methods:**

This international multicentre prospective registry is aimed at collecting information on the indication, timing, technique and outcome of DC and cranioplasty in children. The registry will enrol patients ≤ 16 years of age at the time of surgery, irrespective of the underlying medical condition. The study design comprises four obligatory entry points as a core dataset, with an unlimited number of further follow-up entry points to allow documentation until adolescence or adulthood. Study centres should commit to complete data entry and long-term follow-up.

**Results:**

Data collection will be performed via a web-based portal (homepage: www.pedccr.com) in a central anonymised database after local ethics board approval. An ESPN steering committee will monitor the project’s progress, coordinate analyses of data and presentation of results at conferences and in publications on behalf of the study group.

**Conclusion:**

The registry aims to define predictors for optimal medical care and patient-centred treatment outcomes. The ultimate goal of the registry is to generate results that are so relevant to be directly transferred into clinical practice to enhance treatment protocols.

## Introduction

Decompressive craniectomy (DC) is part of the armamentarium to control critically raised intracranial pressure (ICP) occurring at different stages after severe cerebral insults [1] : Primary DC is used to treat patients with significant space-occupying lesions, in whom the risk of evolving brain edema is high. Secondary or delayed DC is usually considered as a final step if intracranial hypertension becomes refractory to conservative measures. In addition to the implications of primary and secondary brain injury itself, the limitations, inherent risks and complications of DC and also of subsequent cranioplasty have to be taken into account.

Looking at the adult age group, several studies with high-quality methodology have been or are being conducted: A randomised controlled trial (RCT) investigating the role of primary DC (RESCUE-ASDH) has finished enrolment and the final results are not yet published (ISRCTN87370545). Concerning secondary DC for refractory intracranial hypertension (> 25 mmHg) due to severe traumatic brain injury (TBI), the RESCUEicp trial indicated a lower mortality compared to conservative management [2]. The similar DECRA trial had a lower ICP threshold (> 20 mmHg) and demonstrated reduced mortality but more unfavourable outcomes after DC [3]. For malignant ischemic stroke, several RCTs (including HAMLET, DECIMAL, DESTINY I and II) proved a significant reduction of mortality, although DC renders a relevant subgroup with moderately severe disability [4–7].

In contrast, such high-level evidence is not available in the paediatric age group, where the large majority of previous publications are retrospective and monocentric [8–18]. For a recent review on DC in paediatric TBI, Ardissino et al. screened 212 studies, but only 12 ultimately qualified for systematic comparison [19]. The authors concluded that DC reduces mortality and may improve functional outcome, but they also highlighted significant knowledge gaps. Results for DC in paediatric ischemic stroke are limited to case series and anecdotal case reports [20].

Regarding cranioplasty, the level of evidence is low for all age groups. Klieverik et al. recently screened 393 publications on paediatric cranioplasty and ultimately included 24 articles in their systematic review [21]. They concluded that both autologous and alloplastic cranioplasty appeared to be associated with relevant complication rates, with the problem of aseptic bone flap resorption having a pronounced impact on the paediatric cohort. Beyond this, no reliable conclusions were possible and the authors emphasised the relevance of large prospective cohort studies. To improve the evidence base in adults, two prospective multicentre registries are actively recruiting patients ≥ 18 years of age in Europe (UKCRR in the UK and GCRR in Germany, Austria and Switzerland) [22, 23].

The recent efforts of both Ardissino et al. and Klieverik et al. highlight the problems encountered in paediatric DC and cranioplasty [19, 21] : The pooling of published results is hindered by heterogeneous data elements and by missing information. Few studies provide long-term information spanning craniectomy and cranioplasty, although both operations are closely related and relevant to the overall morbidity and outcome of individual patients. If we attempt to fill these evidence gaps with extrapolation of study results obtained in the adult age group, there is a significant caveat: Highly relevant differences in anatomy and physiology are described between adults and children and even within the paediatric age spectrum [24, 25]. Additionally, a fixed point of outcome assessment as used in adults (in virtually all RCTs after 6 and/or 12 months) does not adequately reflect the impact of injuries and treatments on the developing child’s brain. Children require longitudinal observation over many years with age-adjusted outcome measures.

To this point, it should have become clear that the field of paediatric DC and cranioplasty requires significant research activity. However, to further justify such efforts, the relevance of the field has to be taken into account as well. In the paediatric age group, the main cause of severe acute primary and secondary brain injury with consecutive intracranial hypertension and need for decompressive craniectomy (and thus later cranioplasty) is TBI. Therefore, the best epidemiological and health-economic data is available for this condition: 30% of all TBI cases occur in patients under the age of 16, of which approximately 10% suffer moderate or severe TBI [26]. The financial burden of TBI is significant, with estimated annual costs for TBI-related hospitalisation of children in the USA of more than $ 1 billion [27]. With regard to medical outcomes, it is assumed that 30% of children do not survive severe TBI despite DC [14, 28]. Among survivors a good outcome can be expected in 60–90% depending on the type of initial cerebral insult [28, 29]. In addition to the sequelae of the insult itself, the risks of DC and cranioplasty (especially CSF disorders, infections and resorption of autologous bone flaps with the need for revision surgery) need to be taken into account [13, 30–34]. The field is therefore highly relevant for the individual child and, not least due to associated health-care costs, also to society.

The initial proposal for this study was presented at the ESPN Consensus Conference 2019 in Paris. At this conference, a consensus was reached to establish a multicentric registry under the auspices of ESPN. Achieving optimal outcomes after severe insults to the child’s brain is of utmost importance and the ESPN pedCCR will significantly contribute towards this aim.

## Aims and objectives

After reaching consensus to establish a multicentre, prospective, registry under the auspices of ESPN, an initial international steering committee was formed to formulate the study goals and generate a proposal for a study protocol, which was subsequently ratified by the ESPN board.

The primary objective of this study is a detailed systematic assessment of DC and cranioplasty in children (defined as patients ≤ 16 years of age at the time of surgery) with regard to indication, timing, technique and outcomes, irrespective of underlying disease and with a minimum follow-up of 24 months after cranioplasty. The secondary objective is the comparison of treatment strategies and identification of predictors for optimal outcomes of DC and cranioplasty. This study will generate an international multicentric, prospectively collected data set to achieve these objectives.

Such systematic and high-quality data collection and analysis will improve the evidence base and thus medical care in several specific aspects. The concept of the study is characterised by patient orientation: Based on a large, prospective patient series, we aim for identifying risk factors as well as optimal and suboptimal approaches. This effort may ultimately reduce the complication rate, thereby increasing patient safety and optimizing outcome. The study protocol explicitly includes the long-term course and health-related quality of life (using KIDSCREEN-10) in age-dependent self or external assessment, in addition to the King’s Outcome Scale for Childhood Head Injury (KOSCHI) [35, 36]. The latter scale is validated for TBI, but can be applied to other conditions similar to the Glasgow Outcome Scale.

A systematic analysis and comparison of different cranioplasty materials and techniques will deliver further knowledge in order to optimise quality of care and cost-effectiveness in this critical phase after TBI, as cranioplasty carries significant short- and long-term risks in children [34]. Ultimately, based on the data collected, revision surgery could be avoided and implants with the best cost–benefit ratio could be identified. Additionally, the optimal timing of cranioplasty will be analysed, as there is currently conflicting data on early versus delayed cranial reconstruction [37–40]. A further strength of the registry is its focus on specific technical details from a paediatric neurosurgical perspective, which are often impossible to be reconstructed retrospectively from operation notes.

### Study design

**Fig. 1:**
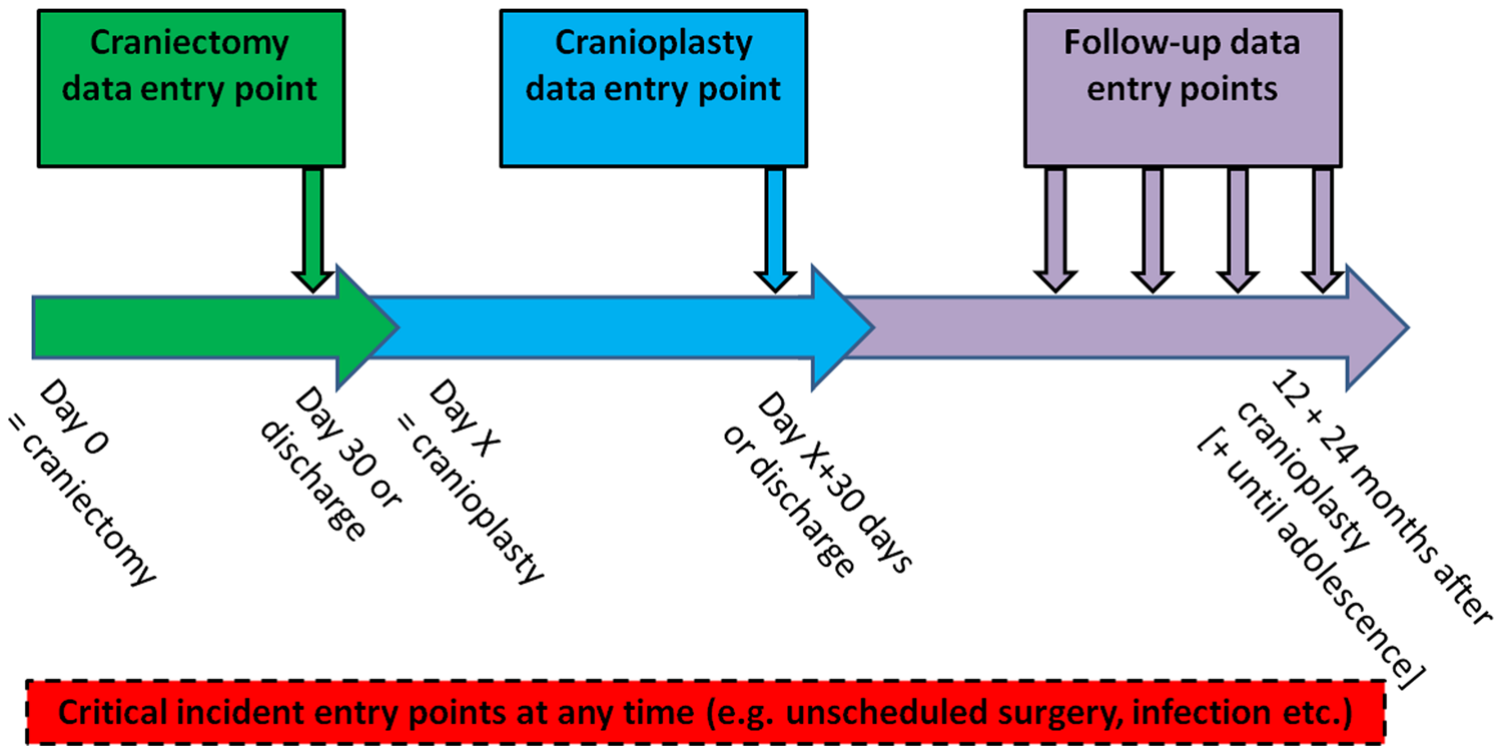
Illustration of the study design, with 4 obligatory data entry points for a complete core data set

This is a multicentre, prospective, registry. Patients ≤ 16 years of age at the time of surgery can be included after informed consent as detailed below, irrespective of underlying disease (i.e. indication for DC). In surviving patients, the study centres are committed to contributing data on subsequent cranioplasty as well as a minimum follow-up of 24 months after cranioplasty (Fig. 1). Further follow-up until adolescence or adulthood is encouraged.

The criteria applying for patient enrolment into the registry were kept simple as a result of the low incidence of DC in children and to actively encourage recruitment (Table 1).

**Table 1:** Overview of inclusion and exclusion criteria

Inclusion criteria	Exclusion criteria
- Age ≤ 16 years at the time of surgery	- Cranioplasty for conditions other than DC (e.g. congenital skull defects)
- Any type of underlying cerebral insult	
- Informed consent obtained	
- Ability to provide follow-up of at least 24 months after cranioplasty	

Based on retrospective data and an exploratory review of the literature, we estimate an annual recruitment of 2 to 5 patients per centre. The experience of the German Cranioplasty Registry for adult patients (GCRR) has shown that approximately 10 national centres can be expected to participate, depending on the size of the country [22]. Sample size justification is based on a minimal assumption of 20 contributing centres for the ESPN pedCCR, with mean enrolment of 3.5 patients per year. The registry could therefore have an annual recruitment of 70 patients.

Data entry will be web-based and the protocol is optimised towards efficiency and low resource requirements for the contributing centres in order to encourage active and lasting enrolment, complete data entries and sufficient follow-up. A complete minimal data set for a patient surviving after DC would include four data entry points, i.e. forms for DC Module, Cranioplasty Module and two Routine Follow-up Modules at 12 and 24 months after cranioplasty, respectively (Fig. 1). The data elements for each module are partially based on the National Institute of Neurological Disorders and Stroke (NINDS) Common Data Elements (https://commondataelements.ninds.gov). Additionally, several validated scores and measures are explicitly or implicitly contained within the forms. The forms for each module are outlined below:

### Decompressive craniectomy module

- 48 items
- Paediatric GCS prior to DC and at discharge or day 30 after DC [41]
- Paediatric Risk of Mortality Score (PRISM) within 4 h after admission [42]
- Rotterdam CT Score [43]
- KOSCHI at discharge or day 30 after DC [35]

### Cranioplasty module

- 26 items
- Paediatric GCS prior to cranioplasty and at discharge or day 30 after cranioplasty
- KOSCHI prior to cranioplasty and at discharge or day 30 after cranioplasty
- KIDSCREEN-10 prior to cranioplasty [36]

### Routine follow-up module

- 10 items
- Paediatric GCS
- KOSCHI
- KIDSCREEN-10
- Oulo Resorption Score (if bone flap resorption observed on imaging) [44]

### Incident reporting module

- 13 items
- Paediatric GCS
- KOSCHI
- KIDSCREEN-10
- Oulo Resorption Score (if bone flap resorption observed on imaging)

### Data management and statistical analysis

**Fig. 2:**
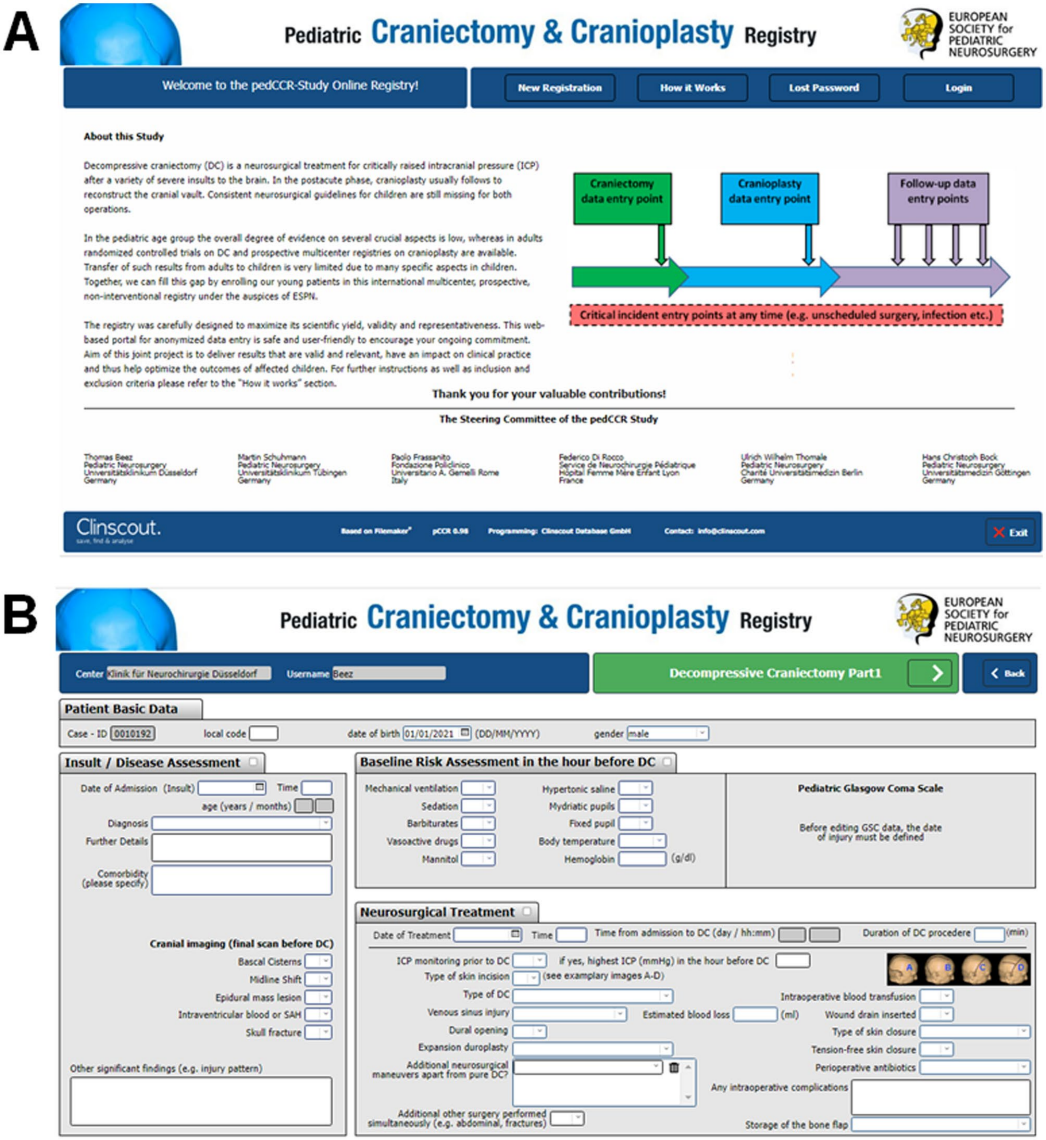
Representative screen shots of the pedCCR database — **A** homepage (homepage: www.pedccr.com) and **B** data entry form for DC

A web-based database using Filemaker^®^ software has been designed to allow password-protected data entry (Fig. 2), similar to the system successfully used for the TROPHY registry [45, 46]. The central server is physically located and professionally hosted in Europe. Data transfer between the user and the study server is encrypted (SSL coding) to assure data privacy. Access to the online registry application is provided via the study homepage: www.pedccr.com.

Patients will be pseudonymised (consecutive numbers) locally by the respective centre. The central data collection will then be done anonymously, i.e. the central database itself does not contain any identifying patient information and the pseudonymisation key will be securely kept at the local centre.

As this is a prospective registry without a limited study period, data collection will be ongoing and no endpoints were predefined. Regular audits will be performed to ensure data quality and integrity. Data analysis will be performed with descriptive statistics. Based on results from the previous literature, the following statistical assumptions regarding relevant clinical variables were made: 30-day-complication rate after DC — 40%; good outcome after DC — 50%; mortality after DC — 30% and autologous bone flap resorption rate — 80%. With the aim of a confidence level of 90% and an error margin of less than ± 10%, an analysis will be carried out for *N* = 100 included cases. To compare the complication rate between autologous versus allogeneic cranioplasty, an evaluation of *N* = 150 cases per cranioplasty modality will be carried out in view of the complication rates from the literature of 33% versus 14%.

### Ethics and informed consent

The study will be carried out in accordance with the principles of the Declaration of Helsinki in the revised version of 2013. The study protocol has been approved by the ethical review board at Heinrich-Heine-University, Düsseldorf, Germany (study number 2021–1653). Each centre will need to have obtained a positive local ethics vote before beginning enrolment. The study protocol and consent forms in German and English will be available for download on the study homepage upon user registration. Since we are including underage subjects, the legal representatives or the carer must provide written consent. If the minor is able to understand the nature of the study, his/her written consent is also required. An age-appropriate adapted patient information leaflet and consent form will be provided. The individual patient data can be deleted completely and irretrievably at any time upon the patient’s request, without giving reasons.

## Conclusions

The lack of high-quality data and thus the low degree of evidence on which treatment decisions can be based with regard to DC and cranioplasty in children became evident during the ESPN Consensus Conference 2019 in Paris. Consensus was reached to create this registry as an important way to systematically collect real-world experiences in the field and analyse and compare treatment approaches across paediatric neurosurgical centres. We believe that this “science of practice” approach will achieve a high degree of internal and external validity and answer important questions and stimulate further research. Contribution and collaboration at all levels, including optimizing the study design, is highly appreciated to advance this project together.
